# Dissecting microsatellite instability in colorectal cancer: one size does not fit all

**DOI:** 10.1186/s13073-017-0438-9

**Published:** 2017-05-24

**Authors:** Robert M. Samstein, Timothy A. Chan

**Affiliations:** 1 Department of Radiation Oncology, Memorial Sloan Kettering Cancer Center, New York, NY 10065 USA; 2 Human Oncology and Pathogenesis Program, Memorial Sloan Kettering Cancer Center, New York, NY 10065 USA; 3 Immunogenomics and Precision Oncology Platform, Memorial Sloan Kettering Cancer C, New York, NY 10065 USA

## Abstract

Microsatellite instability (MSI) marks distinct subsets of tumors in many cancer types and is caused by mutations in genes required for mismatch repair. A recent report analyses the molecular foundations of MSI-positive colorectal cancers and reveals substantial molecular heterogeneity, which might have consequences for the potential use of immunotherapy in MSI-positive cancers.

See related research by Sveen et al. 10.1186/s13073-017-0434-0

## Microsatellite instability in cancer

Hereditary patterns of colon cancer were recognized as early as the 1960s by Henry Lynch. He described Lynch Syndrome, or hereditary non-polyposis colorectal cancer, which is characterized by an autosomal-dominant inheritance pattern and a marked propensity to develop cancers, including colorectal cancer. However, the mechanistic understanding that the tumors of these patients had high numbers of somatic mutations in regions of short tandem repeats or microsatellites and the identification of the causative genetic alterations came decades later [1].

Microsatellite instability (MSI), either due to inherited germline mutations of mismatch repair (MMR) genes or epigenetic inactivation of these genes, is found in approximately 15% of stage II to III colorectal cancers (CRCs) [2]. MMR deficiency leads to a buildup of base-pair mismatches and slippage events over time, causing accumulation of very high numbers of somatic mutations. MSI-positive tumors are present in many other types of cancers in addition to CRCs, including endometrial, gastric, ovarian, gallbladder, prostate, and gliomas [3, 4].

## MSI in CRC prognostics and therapeutic response

The prognostic significance of MSI in CRC has been demonstrated in numerous studies. Decreased rates of recurrence and metastasis, and increased survival occurs in patients whose tumors harbor MMR deficiency in comparison with MMR-proficient tumors [5]. In addition, MSI has been associated with resistance to 5-fluorouracil (5-FU)-based chemotherapy in preclinical models, and clinical trials have demonstrated limited benefit of chemotherapy in the adjuvant setting, particularly in stage II patients [5]. National Comprehensive Cancer Network (NCCN) guidelines recommend MSI testing in all stage II patients as well as screening for Lynch syndrome in patients younger than 70 years of age at diagnosis.

Whereas typical non-MSI tumors may have a hundred or so mutations in the exome, MSI-positive CRCs characteristically have thousands of mutations throughout the coding regions of tumor cells. These tumors are also often characterized by increased numbers of tumor-infiltrating lymphocytes (TILs), including cytotoxic T cells—thought to be a response to the increased number of neoantigens produced by the tumor resulting from the high mutational load. The neoantigens are presented in the context of major histocompatibility complexes (MHCs) and are recognized by the host immune system as foreign [6]. In order to compensate for this inflammation, MSI-positive tumors often upregulate molecules that allow cells to evade the immune system, such as the immune-checkpoint protein PD-L1 [7]. In a promising phase I trial, treatment-refractory MSI-positive tumors demonstrated dramatic responses to PD-1 blockade with the monoclonal antibody pembrolizumab, showing that reversal of these inhibitory pathways could lead to durable immune-mediated tumor control by reactivation of preexisting, exhausted TILs [8]. Numerous phase II and III studies are currently underway to elucidate further the efficacy of immune-checkpoint blockade therapy in MSI-positive CRCs and other cancer types [3]. However, one outstanding question concerns what underlies the observed phenotypic heterogeneity between MSI-positive tumors.

## New insights into the molecular heterogeneity of MSI-positive CRC

In this issue of *Genome Medicine*, Sveen and colleagues [9] analyzed over 300 MSI-positive CRCs from several sources: two Norwegian studies, the British VICTOR trial, The Cancer Genome Atlas, and a multicenter French cohort. A comprehensive analysis was performed on the datasets, which included somatic mutational analysis, clonality analysis, neoantigen load analysis, and gene expression analysis for immune infiltration and consensus molecular subtypes (CMSs). Frequent mutations were identified in several relevant genes, including *CRTC1* (CREB-regulated transcription coactivator 1)*, CCND1/BCL1* (G1/S-specific cyclin-D1)*, PTCH1* (protein patched homolog 1), and *JAK1* (tyrosine-protein kinase JAK1), although many were subclonal (present in only a small fraction of cells), consistent with significant heterogeneity within the tumor. *JAK1* loss-of-function mutations were found in several cohorts, with a prevalence in MSI-positive CRCs of 20%, although mutations were primarily heterozygous. Interestingly, mutated tumors were associated with upregulation of genes associated with resistance to anti-PD1 treatment. However, the heterozygous nature of these mutations and positive association with disease outcomes seen in this study are inconsistent with previous reports, including a recent study reporting association of *JAK1* mutations with resistance to PD-1 blockade in a small cohort of patients [10]. It is possible that, although heterozygous *JAK1* mutations might impart a better prognosis in the absence of immune-checkpoint blockade therapy, homozygous *JAK1* mutation results in a resistance to antibodies against PD1 *in patients treated with immunotherapy*. Further studies are thus required to resolve the ultimate biological effect of these mutations.

As expected, Sveen and colleagues observed increased neoantigen load accompanying increasing mutational load even within these MSI-positive tumors. Immune infiltration, as measured by gene expression, was associated with the CMS1 gene-expression subtype but was not associated with increased mutational burden. Here, it is possible that specific antigens are present in this group of tumors or they have a microenvironment that is more permissive to T-cell infiltration. *JAK1* mutations and the CMS1 subtype were both significantly associated with improved survival.

## Future directions and clinical implications

These results represent the largest analysis devoted to MSI-positive CRCs reported to date and offer key insights into our understanding of MSI and potential determinants of the response to immunotherapies. An important conclusion of this study is that not all MSI-positive tumors are the same, as there exist markedly different immunophenotypes and clinical behaviors within this subgroup of tumors. Considering that mutational load and microsatellite instability are soon to be used in the selection and/or prioritization of patients to receive immune-checkpoint inhibitors, a greater understanding of the heterogeneity within hypermutated tumor subsets and operative drivers of resistance is crucial for their success as clinical biomarkers. This study provides a nice window into the heterogeneity that must be accounted for as researchers optimize the use of immunotherapeutics for the treatment of human cancers.

## Abbreviations

CMS: Consensus molecular subtype
CRC: Colorectal cancer
MHC: Major histocompatibility complex
MMR: Mismatch repair
MSI: Microsatellite instability
TIL: Tumor-infiltrating lymphocyte

## References

[1] Lynch HT, Snyder CL, Shaw TG, Heinen CD, Hitchins MP (2015). Milestones of Lynch syndrome: 1895–2015. Nat Rev Cancer.

[2] André T, de Gramont A, Vernerey D, Chibaudel B, Bonnetain F, Tijeras-Raballand A (2015). Adjuvant fluorouracil, leucovorin, and oxaliplatin in stage II to III colon cancer: updated 10-year survival and outcomes according to BRAF mutation and mismatch repair status of the MOSAIC Study. J Clin Oncol.

[3] Gelsomino F, Barbolini M, Spallanzani A, Pugliese G, Cascinu S (2016). The evolving role of microsatellite instability in colorectal cancer: a review. Cancer Treat Rev.

[4] Lee V, Murphy A, Le DT, Diaz LA (2016). Mismatch repair deficiency and response to immune checkpoint blockade. Oncologist.

[5] Sinicrope FA, Foster NR, Thibodeau SN, Marsoni S, Monges G, Labianca R (2011). DNA mismatch repair status and colon cancer recurrence and survival in clinical trials of 5-fluorouracil-based adjuvant therapy. J Natl Cancer Inst.

[6] Deschoolmeester V, Baay M, Van Marck E, Weyler J, Vermeulen P, Lardon F (2010). Tumor infiltrating lymphocytes: an intriguing player in the survival of colorectal cancer patients. BMC Immunol.

[7] Llosa NJ, Cruise M, Tam A, Wicks EC, Hechenbleikner EM, Taube JM (2015). The vigorous immune microenvironment of microsatellite unstable colon cancer is balanced by multiple counter-inhibitory checkpoints. Cancer Discov.

[8] Le DT, Uram JN, Wang H, Bartlett BR, Kemberling H, Eyring AD (2015). PD-1 blockade in tumors with mismatch-repair deficiency. N Engl J Med.

[9] Sveen A, Johannessen B, Tengs T, Danielsen SA, Eilertsen IA, Lind GE, et al. Multilevel genomics of colorectal cancers with microsatellite instability—clinical impact of JAK1 mutations and consensus molecular subtype 1. Genome Med. 2017. doi:10.1186/s13073-017-0434-0. 10.1186/s13073-017-0434-0 PMC5442873 28539123

[10] Zaretsky JM, Garcia-Diaz A, Shin DS, Escuin-Ordinas H, Hugo W, Hu-Lieskovan S (2016). Mutations associated with acquired resistance to PD-1 blockade in melanoma. N Engl J Med.

